# Temporal Bone Pneumatization and Pulsatile Tinnitus Caused by Sigmoid Sinus Diverticulum and/or Dehiscence

**DOI:** 10.1155/2015/970613

**Published:** 2015-10-25

**Authors:** Liu Wenjuan, Liu Zhaohui, Zheng Ning, Zhao Pengfei, Dong Cheng, Wang Zhenchang

**Affiliations:** ^1^Department of Radiology, Beijing Friendship Hospital, Capital Medical University, Beijing 100050, China; ^2^Department of Radiology, Jining No. 1 People's Hospital, Shandong 272002, China; ^3^Department of Radiology, Beijing Tongren Hospital, Capital Medical University, Beijing 100730, China

## Abstract

*Background*. Although air cells within temporal bone may play an important role in the transmission of pulsatile tinnitus (PT) noise, it has not been studied systematically. *Purpose*. To evaluate the difference in temporal bone pneumatization between PT patients with sigmoid sinus diverticulum and/or dehiscence (SSDD) and healthy people. *Material and Methods*. A total of 199 unilateral persistent PT patients with SSDD and 302 control subjects underwent dual-phase contrast-enhanced CT (DP-CECT), to assess the grade of temporal bone pneumatization in each ear. *Results*. In the bilateral temporal bone of 302 controls, 16 ears were grade I, 53 were grade II, 141 were grade III, and 394 were grade IV. Among the affected ears of 199 PT cases, 1 ear was grade I, 18 were grade II, 53 were grade III, and 127 were grade IV. There was no significant difference in the pneumatization grade between the affected PT ear and either ear in the healthy subjects (*p* > 0.05).  *Conclusion*. Although air cells within the temporal bone are an important factor in the occurrence of PT, its severity does not differ significantly from the pneumatization of healthy people.

## 1. Introduction

Tinnitus is a common otologic symptom affecting 30% of the population worldwide [1]. Approximately 4% of patients have pulsatile tinnitus (PT), defined as the perception of somatosounds synchronized with the pulse in the absence of an external acoustic stimulus [2]. The psychological impact of PT on many patients is so severe that it can lead to depression or even suicide [3]. Although PT has numerous causes, sigmoid sinus diverticulum and/or dehiscence (SSDD) is the most frequent and treatable cause [4–10]. Some studies report that SSDD is the cause of PT in up to 20% of cases [5, 8, 9].

The mechanism of PT caused by SSDD remains unclear. Many studies have suggested that sound in the sigmoid sinus or vibration of the sigmoid wall caused by blood flow is transmitted to the inner ear through the dehiscent sigmoid sinus plate and air cells within the temporal bone, which is ultimately sensed as PT [8, 10, 11]. Thus, the air cells within the temporal bone may be an important contributor to PT occurrence. However, the magnitude of temporal bone pneumatization necessary to trigger PT and the difference between PT patients and healthy people, which is critical to the etiological diagnosis and may affect therapeutic planning, is still debated at present. Some studies suggest that the diffused pneumatization in temporal bone and the pneumatization in patients with PT caused by the internal carotid artery (ICA) were greater than those observed in healthy people and may be an important contributor to PT. Sözen et al. [12] evaluated the relationship between subjective PT and petrous bone pneumatization by comparing 25 PT patients with healthy individuals. They detected petrous bone pneumatization in 22 (68.8%) of 32 ears with subjective PT, which was statistically higher than the prevalence in control subjects (24%). By contrast, a different study found that 80% of PT patients with sigmoid sinus diverticulum exhibited hyperpneumatization or good pneumatization of the temporal bone, while 20% of their patient sample exhibited moderate pneumatization of the temporal bone [11].

The aim of this study was to evaluate the temporal bone pneumatization in PT caused by SSDD by comparing patients diagnosed with PT and healthy control subjects. We hypothesized that the temporal bone pneumatization of the PT group was greater than the pneumatization in the control subjects.

## 2. Materials and Methods

### 2.1. Subjects

The Hospital Institutional Review Board for Human Subjects Research approved this study. We evaluated the pneumatization of temporal bone in the unilateral PT group and control group. The PT group included 199 SSDD patients comprising 18 male and 181 female diagnosed with unilateral PT between May 2008 and January 2013. Sixty-five patients had left PT, and 134 had right PT. The duration of PT was 3 months to 36 years. All patients were diagnosed with SSDD using dual-phase contrast-enhanced computed tomography (DP-CECT) of the temporal bone, and other causes of PT, which was proven by imaging examination and other clinical examination, such as aberrant internal carotid artery, abnormal emissary vein, dural arteriovenous fistula, benign intracranial hypertension, carotid atherosclerosis, paraganglioma, high-riding or dehiscent jugular bulb, or otosclerosis, were excluded. Thirty-eight patients suffering severe and continuous tinnitus underwent surgery of sinus wall reconstruction. Among them, the PT resolved completely in 29 patients and decreased significantly in the remaining 9 patients. Among 937 consecutive patients with orbital tumors, paranasal sinus tumors, or orbital trauma undergoing DP-CECT between May 2008 and January 2013 who did not have a history of tinnitus, we identified 302 patients (209 male and 93 female) as controls after excluding patients undergoing brain surgery or those with low-quality images, temporal bone fracture, and other ear pathologies.

### 2.2. Imaging Method

DP-CECT was performed in all patients using a 64-slice multidetector CT (Brilliance 64; Philips, Best, Netherlands) at the following parameters: 120 kVp, 300 mA, detector collimation 64 × 0.625 mm, rotation time 0.75 s, pitch 0.89 : 1, matrix 512 × 512, and field-of-view (FOV) 22 × 22 cm. The scan range was from the vertex to the sixth cervical vertebrae. Iodinated nonionic contrast agent (Iopamidol (370 mg iodine/mL); Bracco, Shanghai, China) was administered at 5 mL/s using an electric power injector. The contrast agent was dosed at 1.5 mL/kg according to the patient's weight in all subjects. The ascending aorta served as the trigger point, the trigger area measured 200 mm^2^, and the trigger threshold was set at 150 HU. The arterial phase was triggered by the Bolus-Tracking program (Trigger Bolus software; Philips, Best, Netherlands) after administering the contrast agent and performed in the cephalocaudal direction. The arterial phase time ranged from 8 to 12 s. The venous phase scan was performed in the opposite direction after a fixed 8 s delay. All arterial phase images were reconstructed by standard algorithms, and standard and bone algorithms were used in all venous phase images.

### 2.3. Image Interpretation

The CT images were reviewed by two radiologists (LZH and LWJ, with 13 and 11 years of experience, resp.), and the findings were determined by consensus. SSDD was diagnosed according to the following previously described criteria: incomplete thin bone surrounding the sigmoid sinus and/or a diverticulum entering the mastoid bone [5, 6, 13].

The temporal bone pneumatization was classified according to the method described by Han et al. [14]. The sigmoid sinus was the designated reference for evaluation. On the image in which the malleoincudal complex resembled an ice cream cone-shape, three parallel lines angled 45° anterolaterally were applied so that one line crossed the most anterior point of the sigmoid sinus at its junction with the petrous bone, the second line crossed the most lateral margin along the transverse plane of the sigmoid groove, and the third line crossed the most posterior point of the sigmoid sinus, respectively. The magnitude of temporal bone pneumatization was classified into four categories as follows: grade I (hypopneumatization), the pneumatization remained anteromedial to the line drawn at the most anterior point of the sigmoid sinus; grade II (moderate pneumatization), the pneumatization extended into the space between the two lines at the most anterior and lateral aspects of the sigmoid sinus; grade III (good pneumatization), the pneumatization extended to the space between the two lines at the most lateral and posterior aspects of the sigmoid sinus; and grade IV (hyperpneumatization), the pneumatization extended posterolaterally beyond the line drawn at the most posterior point of the sigmoid sinus.

### 2.4. Data Analysis and Statistics

All statistical analyses were performed using statistical software (SPSS, version 16.0; SPSS, Chicago, IL USA), and a *p* value less than 0.05 was considered statistically significant. The *χ* ^2^ test was used initially to analyze the control group according to laterality and gender. The independent *t*-test was then used to access the PT and control groups according to age. If there were no differences in the pneumatization grade between each lateral side and gender in the control group, and the patient ages could be matched between the two groups, then the *χ* ^2^ test was used to analyze the proportional data between the affected side of the PT patients and the control subjects.

## 3. Results

Tables 1 and 2 list the temporal bone pneumatization grade per lateral side and gender, respectively, in the control group. There was no significant difference according to laterality (*p* = 0.471) or gender (*p* = 0.775).

The PT group included 199 PT patients with a mean age of 40.6 ± 13.8 years (range 17–77 years), and the control group included 302 patients with a mean age of 40.8 ± 11.7 years (range 13–80 years). There was no significant difference in age between the PT and control groups (*p* = 0.865).

As shown in Table 3, there was no significant difference in the pneumatization grade between the affected side in the PT group and either side in the control group (*p* = 0.263).

## 4. Discussion

Our study found that the temporal bone pneumatization grade in patients with PT was identical to the grade in healthy people. Additionally, although sigmoid plate dehiscence has been reported as a cause of PT, we found that it also occurs in some healthy people. Therefore, abnormal blood flow within the sigmoid sinus is the essential factor triggering PT. Only when abnormal sigmoid sinus perfusion, sigmoid plate dehiscence, and normal temporal bone pneumatization coexist will PT potentially occur.

Approximately 90.5% of the temporal bone pneumatization lesions were grades II and IV (good pneumatization and hyperpneumatization), which supports the theory that extensive temporal bone pneumatization favors sound transmission, as suggested by others. Large air cells increase the resonance of sound and serve as an amplifier. Increased transmission of normal perfusion sounds to the cochlea would lead to PT [15, 16]. However, 9.5% of temporal bone pneumatization in the patient population were grades I and II (hypopneumatization and moderate pneumatization). Multiple small air cells in poorly pneumatized temporal bone do not augment the resonance of blood flow vibration in the sigmoid sinus. However, the transmission distance from the sigmoid sinus to the inner ear is shorter than in extensively pneumatized temporal bone and therefore may be sensed as PT.

In our study, DP-CECT was used to examine the detailed anatomic structure and accurately diagnose PT caused by SSDD. DP-CECT includes arterial and venous phases and can demonstrate the status of vessels and temporal bones simultaneously in a single study. Krishnan et al. [17] suggested that DP-CECT can effectively detect arterial, venous, and inner ear causes of PT in a prospective study of 16 PT patients. Han et al. [14] classified the temporal bone pneumatization into grades I–IV based on the sigmoid sinus, which can be used to assess pneumatization with good feasibility. The method used a single CT image and assessed the structure of the sigmoid sinus, which was found to accurately represent pneumatization in the entire temporal bone. In our study, we chose this method to classify the temporal bone pneumatization because it was simple and practical for a large sample analysis.

Notably, our study is a retrospective study, and the difference in temporal bone pneumatization was analyzed between PT patients and healthy subjects. However, the specific mechanism generating the air cells within the temporal bone in PT was not examined and warrants further experimental study.

## 5. Conclusion

The magnitude of temporal bone pneumatization does not significantly differ between PT patients with SSDD and healthy people, which indicates that normal pneumatized temporal bone can potentially meet the criteria of SSDD required to induce PT.

## Figures and Tables

**Table 1:** Comparison of temporal bone pneumatization grade between left and right ears in control group.

Pneumatization grade	Side (number of ears)		Statistical analysis	
	Left	Right	*χ* ^2^	*p*
I	8	8	2.526	0.471
II	22	31		
III	67	74		
IV	205	189		
Total	302	302		

**Table 2:** Comparison of temporal bone pneumatization grade by gender in control group.

Pneumatization grade	Gender (number of ears)		Statistical analysis	
	Male	Female	*χ* ^2^	*p*
I	12	4	1.157	0.775
II	39	14		
III	94	47		
IV	273	121		
Total	418	186		

**Table 3:** Comparison of temporal bone pneumatization grade between the PT and control groups.

Pneumatization grade	Group (number of ears)		Statistical analysis	
	PT side	Control	*χ* ^2^	*p*
I	1	16	3.986	0.263
II	18	53		
III	53	141		
IV	127	394		
Total	199	604		

PT: pulsatile tinnitus.
